# Case report: a R0 resection successfully induced by T-DXd plus PD-1 inhibitor regimen in a primary unresectable stage IIIB NSCLC with *ERBB2*-mutation

**DOI:** 10.1038/s41698-025-00982-x

**Published:** 2025-06-10

**Authors:** Ruijie Zhang, Sida Cheng, Kunkun Sun, Lihong Zhang, Xiang Yan, Fan Yang

**Affiliations:** 1 Department of Thoracic Surgery, Peking University People’s Hospital, Beijing, China; 2 Department of Pathology, Peking University People’s Hospital, Beijing, China; 3 Department of Otorhinolaryngology, Head and Neck Surgery, Peking University People’s Hospital, Beijing, China

**Keywords:** Non-small-cell lung cancer, Surgical oncology, Targeted therapies

## Abstract

Chemoradiotherapy (CRT) with consolidative immunotherapy(IO) remains the standard care for unresectable stage III NSCLC, yet survival remains suboptimal in molecular subgroups. Emerging targeted therapies offer the potential for converting unresectable cases to resectable status. We present a 54-year-old female with stage IIIB (T1cN3M0) *ERBB2* exon 20-mutated lung adenocarcinoma (PD-L1 50%) and supraclavicular metastasis. After three cycles of *ERBB2*-targeted antibody-drug conjugate combined with programmed death-1 (PD-1) inhibitor, transient Grade 1 interstitial lung disease resolved with steroids. Serial imaging revealed 58% tumor regression, enabling successful R0 resection via VATS lobectomy with lymphadenectomy. Histopathological analysis confirmed complete pathological response in primary and nodal lesions, corroborated by undetectable postoperative minimal residual disease. The patient remained recurrence-free at a 9-month follow-up. This induction targeted-IO strategy enabled surgical conversion in ERBB2-mutant NSCLC with durable remission and acceptable toxicity, warranting investigation in molecular subgroups with limited CRT-IO benefit.

## Introduction

Stage III non-small cell lung cancer (NSCLC) exhibits significant heterogeneity, requiring variable management and multimodal treatment approaches. Patients with stage IIIB disease, such as those with multistation N2 metastases, bulky N2, or N3, are typically deemed unresectable for surgery. The current standard of care for such cases remains concurrent (cCRT)^1^ or sequential chemoradiotherapy (sCRT)^2^ followed by maintenance anti-PD1 therapy. How to achieve surgical conversion for these unresectable patients is a critical step toward curative treatment by enhancing locoregional control. Emerging molecularly targeted therapies offer transformative potential for driver-mutated subgroups^3^. Notably, *ERBB2*-mutant NSCLC (accounting for 2–4% of cases)^4^ demonstrates inherent chemoresistance but responds remarkably to novel antibody-drug conjugate (ADC) like trastuzumab deruxtecan (T-DXd)^5–8^. However, its application in perioperative management remains unexplored.

Therefore, we attempted to introduce T-DXd as a novel approach combined with PD-1 inhibitor in the induction treatment for of one unresectable stage IIIB patient with ERBB2 mutation. The surgical conversion achieved successful surgical conversion and pathological response with acceptable toxicity.

## Results

### Case report

**Fig. 1:**
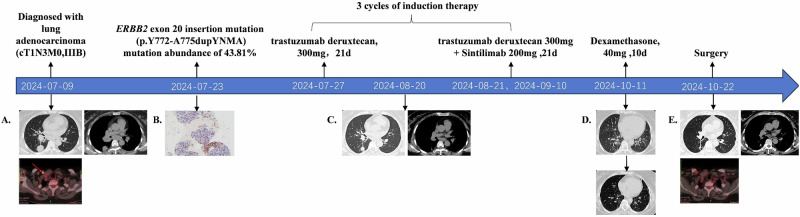
Treatment timeline during induction therapy and subsequent surgery. **A** Positron emission tomography/computed tomography (PET/CT) scan reveals a mass in the right lower lobe, along with enlarged hilar and mediastinal lymph nodes, and FDG uptake in the right supraclavicular lymph node. **B** Next-generation sequencing (NGS) identifies an *ERBB2* p.Y772-A775dupYNMA mutation (43.81% VAF). Immunohistochemical analysis shows PD-L1 expression (22C3, TPS 50%). **C** CT images after one cycle of induction therapy demonstrate a reduction in the size of the tumor lesion. **D** CT images after three cycles of induction therapy show a further reduction in tumor size, with slight interstitial lung disease (Grade 1). Significant remission is observed after a 10-day course of dexamethasone. **E** PET/CT scan shows shrinkage of the tumor lesion and decreased FDG uptake in the right supraclavicular lymph node.

A 54-year-old Chinese female presented to the Peking University People’s Hospital with intermittent cough, sputum production, and shortness of breath, with an Eastern Cooperative Oncology Group performance status of 1. She reported no history of smoking or any notable medical conditions. Positron emission tomography/computed tomography presented a mass of 3.0 cm × 2.5 cm, centrally located in the right lower lobe, accompanied by significantly enlarged hilar, mediastinal (stations 1, 2R, 4R, and 7), and right supraclavicular lymph node with elevated maximum standardized uptake value, and the largest lymph node measuring 3.3 × 2.8 cm (Fig. 1A). Lung adenocarcinoma was pathologically confirmed in stations 4, 7, and 10 by EBUS-TBNA, with PD-L1 TPS 50% and *ERBB2*(human epidermal growth factor receptor 2, *HER2*) p.Y772-A775dupYNMA mutation (Fig. 1B). Following multidisciplinary team (MDT) discussions involving medical oncology, thoracic surgery, radiation oncology, and other relevant specialties, the patient was staged as cT1bN3M0 (IIIB, AJCC 8th), unresectable according to the guidelines.

The standard treatment with definitive chemoradiotherapy followed by immunotherapy consolidation, and the induction therapy followed by potential radical surgery were both considered. Given the patient’s strong desire for surgical resection and full understanding of the potential risks and benefits, the MDT team agreed that surgery might be considered if a favorable response to induction therapy was achieved, which can provide better local control. Also, the definitive radiotherapy followed by immunotherapy can be the backup choice if the induction response is poor. Therefore, the patient initiated induction therapy with T-DXd (300 mg q3w) plus sintilimab (200 mg q3w).

### Subsequent clinical course

After one cycle of T-DXd monotherapy, imaging showed a partial response. The tumor continued to shrink after the subsequent two cycles of combination treatment, achieving a 58% regression rate (Fig. 1C, D). A slight interstitial lung disease (ILD) (Grade 1) was found but recovered quickly with a 10-day course of dexamethasone (40 mg daily), and the surgery was not delayed.

**Fig. 2:**
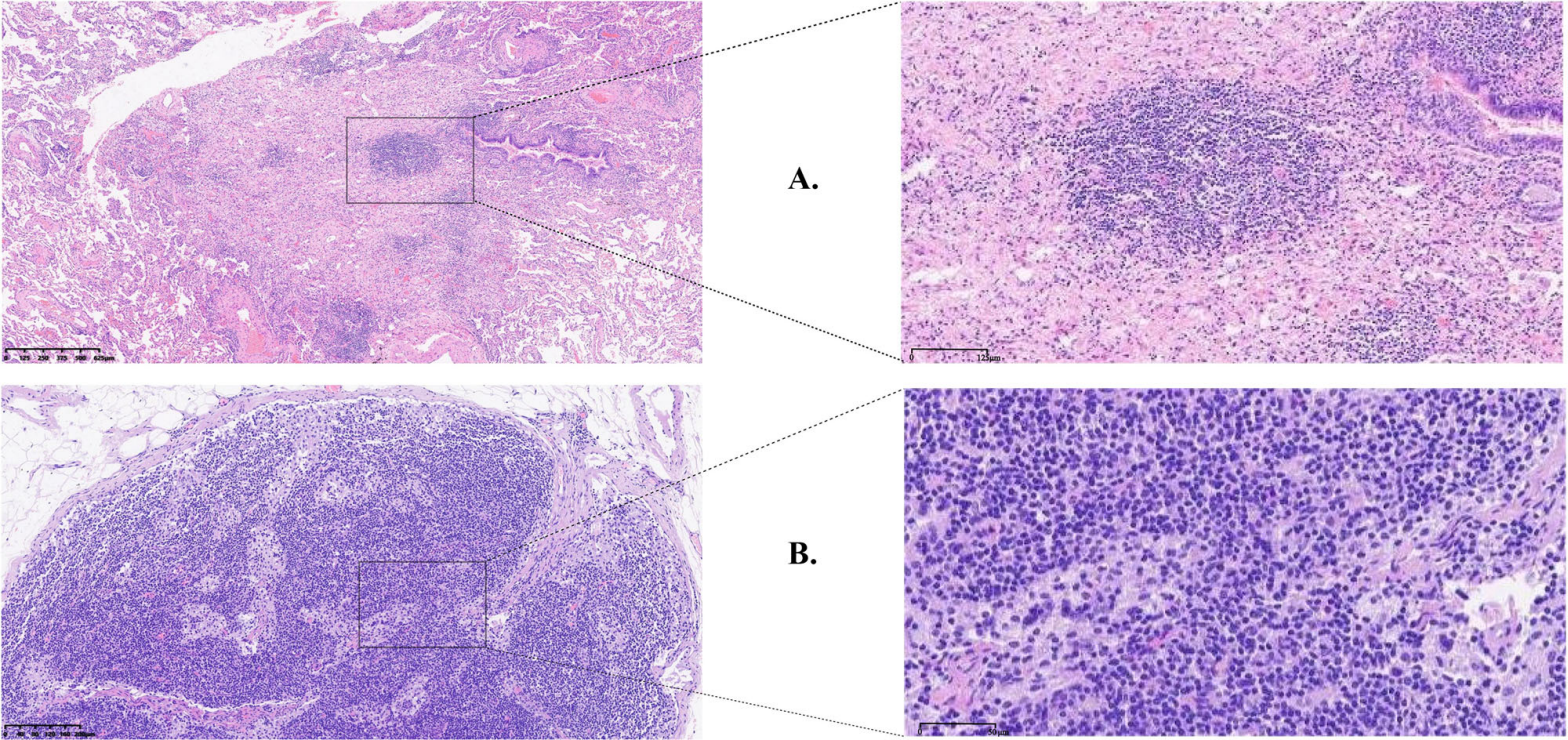
Histological morphology of the primary tumor and the right supraclavicular lymph node. **A** Right lower lobe lung resection specimen: Lung tissue shows focal fibrous tissue proliferation, accompanied by focal lymphocytic and histiocytic infiltration, with areas of necrosis. No residual tumor cells were observed. (HE side, 40×). **B** Right supraclavicular lymph node from zones IV and V: No evidence of cancer metastasis was found. (HE side, 10×).

Video-assisted thoracoscopic surgery (VATS) was performed for a right lower lobectomy, systematic lymph node dissection, and right supraclavicular lymph node dissection six weeks after treatment. The surgery took 120 min, with 20 mL of blood loss, and the patient was discharged after a three-day hospital stay without postoperative complications. The postoperative pathological assessment confirmed a R0 resection and pathological complete response (pCR) without viable tumor (Fig. 2). The postoperative tumor-informed MRD test turned out to be negative.

Based on the patient achieving pCR after surgery and testing negative for MRD, compared to the postoperative maintenance therapy with T-DXd, we decided to opt for regular follow-up. Routine imaging evaluations per three months were suggested during follow-up. Nine months after the surgery, no recurrence or metastasis was observed.

## Discussion

For unresectable stage III NSCLC, surgery conversion could represent a crucial step toward a cancer cure with the assistance of the new anti-tumor drugs. The TRAILBLAZER study^9^ suggests that induction therapy based on immunotherapy significantly improves pathological downstaging rates in unresectable stage III NSCLC. In N3 disease, neoadjuvant therapy achieved 66.7% pathological downstaging and 25% surgical conversion, with all resected patients attaining R0 resection and 26% achieving pCR. This strategy significantly improved median PFS in surgical versus non-surgical patients (29 vs. 14 months, *P* = 0.007). This study expanded the available choices for guiding our therapeutic approach.

Primary consideration should be given to whether an immunotherapy-based strategy should be adopted. In the CheckMate 816 trial^10^, the pCR rate improvement was significant in PD-L1-positive populations treated with nivolumab (TPS < 1%: EFS 14.1%; TPS ≥ 1%: EFS 30.3%; TPS ≥ 50%: EFS 40.0%). In the CheckMate 77 T trial^11^, the improvement in pCR rates among PD-L1-positive populations was even more pronounced (TPS < 1%: EFS 12.9%; TPS ≥ 1%: EFS 35.2%; TPS ≥ 50%: EFS 51.1%). These findings collectively indicate that patients with high PD-L1 expression may derive significant benefits from immunotherapy-based regimens, thereby providing a robust evidence base for selecting immune checkpoint inhibitors in this case.

Notably, this patient exhibited a distinct molecular feature: an *ERBB2* gene mutation. Trastuzumab Deruxtecan, an ADC targeting *ERBB2*, represents a promising therapeutic advancement for this patient population^5^. The DESTINY-Lung trials established T-DXd as a breakthrough therapy for *ERBB2*-mutant NSCLC. In DESTINY-Lung01^6^, T-DXd achieved an objective response rate (ORR) of 55%, median progression-free survival (PFS) of 8.2 months, and median overall survival (OS) of 17.8 months. The subsequent DESTINY-Lung02^7^ study explored dose optimization and found that lower dosing significantly reduced the incidence of complications such as interstitial lung disease (ILD) (26% vs. 6%) while maintaining comparable efficacy. In our case, we adopted a low dose of 5.4 mg/kg, and only a grade 1 adverse event responsive to steroids was observed, further supporting the tolerability of this dosing strategy. Regarding the combination of T-Dxd and PD-1 inhibitors, prior studies in breast cancer have reported a favorable safety profile^12^. Based on this, we employed a sequential combination of T-Dxd and PD-1 inhibitors in this case and performed close monitoring for adverse events. The results showed that the combination was well tolerated in this patient. Notably, the DESTINY-Lung03 trial^8^ has included a cohort evaluating the efficacy and safety of this combination in lung cancer, and we look forward to future data from that study.

At present, there is still no consensus regarding the optimal adjuvant treatment strategy following induction therapy. In this case, the patient achieved pathological complete response (pCR) with MRD-negative status after surgery. Data from previous studies, such as CheckMate 816 and 77T^10,11^, suggest that the benefits of adjuvant immunotherapy are more clearly demonstrated in non-CR populations than in pCR patients. Meanwhile, minimal residual disease (MRD), as an emerging biomarker, has been confirmed by previous studies^13,14^ to be significantly associated with prognosis. Several trials involving neoadjuvant immunotherapy, including AEGEAN^15^, have shown that patients with MRD-negative status after surgery tend to have better survival outcomes compared to those with persistent MRD. However, such approaches should still be used with caution in clinical practice. Based on this context, and after a thorough discussion with the patient, the MDT team decided to proceed with active surveillance, accompanied by regular imaging and MRD monitoring.

We confirmed that surgical conversion after targeted-based induction was feasible in a notable proportion of patients with upfront unresectable disease. Based on previous studies demonstrating improved survival in patients achieving surgical resection, this approach may translate into better long-term outcomes. These positive findings highlight the possible need for a redefinition of unresectable stage III disease in the era of targeted therapy. However, additional clinical data are needed to validate the efficacy and safety of combining T-DXd with PD-1 inhibitors. As a single-case report, the findings are exploratory and require further validation in larger prospective studies.

This exploratory case demonstrates that T-DXd plus PD-1 inhibition enables curative resection in initially unresectable ERBB2-mutant NSCLC without compromising safety. Prospective trials should validate this strategy and optimize its application across molecular subsets.

## Methods

### Patient consent

The studies involving humans were approved by the ethics review committee of Peking University People’s Hospital. The studies were conducted in accordance with the local legislation and institutional requirements. Written informed consent for participation was not required from the participants or the participants’ legal guardians/ next of kin in accordance with the national legislation and institutional requirements. Written informed consent was obtained from the individual(s) for the publication of any potentially identifiable images or data included in this article.

### PD-L1 assessment

Immunohistochemistry staining for PD-L1 was performed using the PD-L1 IHC 22C3 pharmDx assay (Agilent Technologies).

### Next generation sequencing

Targeted next generation sequencing on 654 cancer-related genes (covering SNV, Indel, fusion, and CNV) was performed on fresh core needle biopsy tissue and matched peripheral blood as previously described. The assay utilized a hybridization capture-based next-generation sequencing platform covering coding exons and select introns of 654 genes. Genomic alterations were annotated using an in-house variant interpretation system based on the 2017 AMP-ASCO-CAP guidelines to identify functionally relevant variants. Variants of unknown significance (class III) were excluded from analysis unless a gene demonstrated recurrent alterations (>15% frequency in the study population). TMB was calculated as the total number of nonsynonymous mutations per megabase of sequenced coding regions. Copy number alterations were determined using normalized read depth ratios, with amplifications defined as copy number (CN) ≥ 4.0 (e.g., ERBB2 CN = 4.64, CCNE1 CN = 5.28) and gains as CN ≥ 2.0 but <4.0. Tumor purity and ploidy were integrated into CNV interpretation. Immune biomarkers, including PD-L1 expression (TPS 50%, CPS 60), MSI status (MSS), and HLA-I genotyping (A01:01/24:02, B51:01/15:17, C07:01/14:02), were analyzed separately using orthogonal methods.

### MRD assessment

Circulating tumor DNA (ctDNA) analysis for minimal residual disease (MRD) was performed using the cSMART2.0 technology (Kanghui Biotech), as previously described1,2. Peripheral blood (10 mL) collected in cell-free DNA preservation tubes was centrifuged to isolate plasma. Hybridization capture-based next-generation sequencing targeted 218 cancer-related genes with a median sequencing depth of 62,776× and 100% coverage. Variant calling was optimized for ultra-low-frequency mutations (detection limit: 0.02% variant allele frequency). MRD negativity was defined as the absence of tumor-specific somatic variants (e.g., ERBB2 p.Y772_A775dup, TP53 p.P152S) in plasma.

## Supplementary information

CARE-checklist

## Data Availability

No datasets were generated or analysed during the current study.
